# Functional Outcomes and Morbidity in Pediatric Sepsis Survivors: A Tanzanian Experience

**DOI:** 10.3389/fped.2021.805518

**Published:** 2022-01-17

**Authors:** Sarah A. Lau-Braunhut, Audrey M. Smith, Martina A. Steurer, Brittany L. Murray, Hendry Sawe, Michael A. Matthay, Teri Reynolds, Teresa Bleakly Kortz

**Affiliations:** ^1^Department of Pediatric Critical Care, Banner Children's at Desert Hospital, Mesa, AZ, United States; ^2^Division of Critical Care, Department of Pediatrics, University of California, San Francisco, San Francisco, CA, United States; ^3^Institute for Global Health Sciences, University of California, San Francisco, San Francisco, CA, United States; ^4^Department of Pediatrics and Department of Emergency Medicine, Emory University School of Medicine, Atlanta, GA, United States; ^5^Department of Emergency Medicine, Muhimbili University of Health and Allied Sciences, Dar es Salaam, Tanzania; ^6^Departments of Medicine and Anesthesia, Cardiovascular Research Institute, University of California, San Francisco, San Francisco, CA, United States; ^7^World Health Organization, Geneva, Switzerland

**Keywords:** pediatric sepsis, functional outcome, functional status scale, morbidity, quality of life, LMIC (low-income and middle-income countries), resource-limited and remote setting

## Abstract

Pediatric sepsis remains a significant cause of childhood morbidity and mortality, disproportionately affecting resource-limited settings. As more patients survive, it is paramount that we improve our understanding of post-sepsis morbidity and its impact on functional outcomes. The functional status scale (FSS) is a pediatric validated outcome measure quantifying functional impairment, previously demonstrating decreased function following critical illnesses, including sepsis, in resource-rich settings. However, functional outcomes utilizing the FSS in pediatric sepsis survivors have never been studied in resource-limited settings or in non-critically ill septic children. In a Tanzanian cohort of pediatric sepsis patients, we aimed to evaluate morbidity associated with an acute septic episode using the FSS modified for resource-limited settings. This was a prospective cohort study at an urban referral hospital in Tanzania, including children with sepsis aged 28 days to 14 years old over a 12-month period. The FSS was adapted to the site's available resources. Functional status scale scores were obtained by interviewing guardians both at the time of presentation to determine the child's baseline and at 28-day follow-up. The primary outcome was “decline in functional status,” as defined by a change in FSS score of at least 3. In this cohort, 4.3% of the 1,359 surviving children completing 28-day follow-up had a “decline in functional status.” Conversely, 13.8% of guardians reported that their child was not yet back to their pre-illness state. Three-quarters of children reported as not fully recovered were not identified *via* the FSS as having a decline in functional status. In our cohort of pediatric sepsis patients, we identified a low rate of decline in functional status when using the FSS adapted for resource-limited settings. A higher proportion of children were subjectively identified as not being recovered to baseline. This suggests that the FSS has limitations in this population, despite being adapted for resource-limited settings. Next steps include developing and validating a further revised FSS to better capture patients identified as not recovered but missed by the current FSS.

## Introduction

Sepsis remains one of the leading causes of childhood morbidity, mortality, and utilization of healthcare worldwide (1). Sepsis incidence and sepsis-related mortality disproportionately affect children in low- and middle-income countries (LMICs), with the highest health-related burden of sepsis afflicting sub-Saharan Africa (1, 2). Global sepsis incidence peaks in early childhood, with over half of all sepsis cases worldwide occurring among children and adolescents; in 2017, there were an estimated 48.9 million total cases of sepsis worldwide with 20.3 million cases in children under 5 years of age and 4.9 million cases in those aged 5–19 years (2). Fortunately, despite the high global incidence of sepsis, pediatric sepsis-related mortality has declined consistently over the past several decades (2). However, there continues to be disparate morbidity and mortality outcomes between resource-rich and resource-poor settings, with up to two-thirds of pediatric sepsis-related deaths occurring in sub-Saharan Africa (2–4). Furthermore, given the persistently high incidence of acute sepsis episodes, with more children surviving, it has become paramount that we improve our understanding of post-sepsis morbidity and how to preserve function and quality of life (QOL) in pediatric sepsis survivors.

Assessing the incidence of sepsis in the pediatric population, as well as identifying resulting new morbidity, remains challenging due to the lack of consensus definitions for both. Pediatric sepsis is difficult to define and diagnose, particularly in resource-limited settings. New definitions of sepsis focus on a life-threatening dysregulated host response to infection leading to organ dysfunction (5). However, the scoring systems used to identify organ dysfunction and accurately diagnose sepsis in this manner can be challenging in resource-limited settings. Therefore, an international pediatric sepsis consensus defined sepsis using systemic inflammatory response syndrome (SIRS) in the setting of suspected infection (6), which was then modified for and used in resource-limited settings (7). Additionally, new morbidity is equally difficult to define, with no consensus definition across studies. It is generally thought of as a deviation in status from pre-illness baseline, often described as a new impairment in health, or in either or both functional status and QOL. Prior studies have demonstrated new morbidity in pediatric patients following an acute septic episode or critical illness, often with impairment of both functional status and QOL (8–21). Due to lack of a standardized definition and measurement tool, pediatric morbidity outcome data are highly varied secondary to the use of different assessment tools, various outcomes measures, heterogenous patient populations, and differing durations of follow-up (17, 22). Estimates for development of new morbidity following septic episodes vary widely, ranging from 12 to 50%, primarily assessed in resource-rich settings (1, 9, 10, 14, 16, 18, 19, 23). Multiple factors have been associated with higher risk for morbidity, including source of infection, recent trauma, acute kidney injury, receipt of cardiopulmonary resuscitation (CPR), more severe illness, and longer ICU and hospital stay (10, 14, 16, 19).

Multiple assessment tools have been developed and validated for studying functional outcomes and QOL in pediatric patients (24). The functional status scale (FSS) was created as an age-independent assessment of pediatric functional status for use in large pediatric clinical studies (25, 26). The FSS was designed to establish a functional status outcome measure that was clearly defined, quantitative, rapid to complete, objective, and useful in a variety of inpatient environments (26). The FSS has shown validity and reliability in quantifying functional impairment (26, 27). It is comprised of six domains capturing activities of daily living important to one's overall function: mental status, sensory function, communication, motor function, feeding, and respiratory status (26). Previous studies have shown worsening of functional status in children following critical illnesses, including sepsis cases that required admission to the Pediatric Intensive Care Unit (PICU) (8, 9, 11, 12, 26, 27). A change in cumulative FSS score of ≥3 pre and post-illness has been shown in prior studies to be indicative of significant worsening in functional status (9, 11, 12, 25, 28). However, to our knowledge, functional outcomes in pediatric sepsis survivors have never been studied using the FSS in LMICs, or among septic children treated outside of the PICU setting.

In a cohort of pediatric sepsis patients in Tanzania, our primary objective was to evaluate functional morbidity associated with an acute septic episode using the FSS modified for resource-limited settings. Secondary objectives were delineating which functional domains were most commonly impacted, better characterizing follow-up needs of pediatric sepsis survivors, identifying risk factors for poor functional outcomes, and assessing outcomes using guardians' subjective report of recovery.

## Materials and Methods

### Study Design and Setting

This prospective descriptive cohort study of pediatric patients presenting with sepsis was conducted between July 1, 2016 and June 30, 2017 in the Emergency Medicine Department (EMD) of Muhimbili National Hospital (MNH) located in Dar es Salaam, Tanzania. Muhimbili National Hospital is an urban, tertiary referral hospital with the only public, 24-h, full capacity EMD in Tanzania. This EMD is the department responsible for triaging all patients with acute illness and injury, and treats an estimated 60,000 patients annually, of which approximately 25% are pediatric. The EMD is staffed around-the-clock by Emergency Medicine trained specialists. It is estimated that around 150–200 pediatric patients with sepsis present to the MNH EMD monthly (29, 30).

Resources routinely available in the MNH EMD include cardiorespiratory monitoring, imaging including radiographs and bedside ultrasound, low-flow oxygen, transfusion of blood products, vasoactive and resuscitation medications, bag-mask ventilation, and intubation and mechanical ventilation. In addition to the EMD, MNH has a large pediatrics ward and a high-dependency unit (HDU). There was no dedicated PICU at MNH at the time of study enrollment. Both the general pediatrics ward and the HDU are equipped similarly to the EMD with the ability to administer intermittent medications, intravenous fluids, and perform reassessments including vital signs. However, intubation, mechanical ventilation, and vasoactive infusions are not typically available for pediatric patients outside of the EMD setting (29).

### Patient Population

The study population for this prospective cohort study was comprised of children presenting with an acute episode of sepsis. All pediatric patients presenting to the MNH EMD were screened for inclusion in the study. Inclusion criteria included children aged ≥28 days to 14 years old who presented to the MNH EMD and met criteria for sepsis, as defined by the international pediatric sepsis consensus definitions based on SIRS criteria (6) adapted for resource-limited settings (7). These included a suspected or proven infection and the presence of at least two of the four following criteria: an abnormal temperature (>38 or <36.0C); abnormal heart rate for age including tachycardia or bradycardia (if <1 year old); respiratory insufficiency including tachypnea for age, hypoxia (SpO_2_ <92%), or requiring non-invasive or invasive respiratory support; and being ill-appearing, in distress, or unresponsive. Additional inclusion criteria required guardians to be primarily English or Kiswahili speaking as self-identified. Exclusion criteria included acute trauma and burns, cardiac arrest upon arrival, and inability of guardian to speak Kiswahili or English.

This study was approved by the Institutional Review Boards and Committees on Human Research at both Muhimbili University of Health and Allied Sciences (Ref. No. 2016-03-30/AEC/Vol.X/201) and the University of California, San Francisco (IRB # 16-18977, Ref. No. 161295). In accordance with the Declaration of Helsinki, informed written consent was obtained from guardians prior to study enrollment for all subjects, and assent was obtained from the subject when appropriate.

### Patient Data and Study Measurements

Patient data and study measurements included: patient characteristics [age, sex, weight, mid-upper arm circumference (MUAC), and immunization status], presence of comorbidities [human immunodeficiency virus (HIV), congenital heart disease, cancer, tuberculosis (TB), malaria, and malnutrition], socioeconomic status indicators (parental education levels, insurance status, number of children in the household), severity of illness indicators [vital signs, SIRS criteria on admission, Lambaréné organ dysfunction score (LODS), and level of consciousness using the AVPU score (alert, responsive to verbal stimuli, responsive to painful stimuli, or unresponsive)], time to definitive care (duration of fever and illness), healthcare facilities visited prior to MNH, referral status, prior receipt of antibiotics, previous assessment at a lower-level facility, interventions received, therapies completed in the EMD, relevant laboratory results data [lactate, serum pH, hemoglobin, white blood cell (WBC) count] collected at the time of admission, and outcomes.

Severe malnutrition was defined as severely underweight, and therefore malnourished, if their weight-for-age z-score was < -3.

The LODS was created as a simple and objective severity of illness score and clinical prediction tool for mortality among children ≤ 15 years of age in Africa, both with and without malaria. It is comprised of a four-point scoring system (ordinal scores of 0–3) with one point each for: prostration (presence of at least one of the following dependent on age: inability to breastfeed, sit, stand, or walk unsupported), coma [defined by a Blantyre coma score (BCS) ≤ 2], and deep breathing (31, 32).

The FSS provides an objective measure of dysfunction both by each of its six individual domains (mental status, sensory function, communication, motor function, feeding, and respiratory status), and as a cumulative measure of global dysfunction. Each domain is scored ordinally between 1 “normal” and 5 “very severe dysfunction.” A summative FSS score is then calculated with ranges from 6 to 30, with 6–7 considered “normal function,” 8–9 “mild dysfunction,” 10–15 “moderate dysfunction,” 16–21 “severe dysfunction,” and >21 “very severe dysfunction” (26).

The original FSS was presented to a group of clinical researchers, physicians, nurses, and parents in Tanzania and subsequently adapted for this study based on locally available resources (Table 1). Research staff obtained FSS scores by interviewing guardians at two time points: (1) at the time of presentation to determine the child's baseline functional status (functional state prior to the current acute illness), and (2) at the 28-day follow-up, 4 weeks after initial presentation. Each FSS domain was first scored ordinally between 1 and 5, as previously defined, and then revised to a summative FSS scaled score from 6 to 29 (26). Research personnel had the opportunity to enter guardian descriptors of the child's status in each functional domain. For subjects with descriptors available, each domain score was reviewed for accuracy and recategorized as appropriate.

**Table 1:** Functional status scale (FSS) adapted for resource-poor settings (scale 6–29).

**FSS domain**	**Normal (Score = 1)**	**Mild dysfunction (score = 2)**	**Moderate dysfunction (score = 3)**	**Severe dysfunction (score = 4)**	**Very severe dysfunction (score = 5)**
Mental status	Normal sleep/wake; appropriate responsiveness	Sleepy but arousable to noise, touch, movement, and/or periods of social non-responsiveness and/or insomnia^a^	Lethargic and/or irritable	Minimal arousal to stimuli	Unresponsive, coma, and/or vegetative state
Sensory functioning	Intact hearing, vision, response to touch	Suspected hearing or vision loss	Not reactive to auditory stimuli OR to visual stimuli	Not reactive to auditory stimuli AND to visual stimuli	Abnormal responses to pain or touch
Communication	Appropriate vocalizations, interactive facial expressiveness, or gestures	Diminished vocalization, facial expression, and/or social responsiveness	Absence of attention-getting behavior	No demonstration of discomfort	Absence of communication
Motor functioning	Coordinated movements, normal muscle control, awareness of action	1 limb functionally impaired	>2 limbs functionally impaired	Poor head control	Diffuse spasticity, paralysis, or posturing
Feeding	All food taken by mouth with age-appropriate help	Cannot eat by mouth without frequent coughing or sputtering^b^	Needs more help with feeding than other children his or her age (ex. pureed foods for any child over 12 months of age)^c^	Receives formula through a feeding tube^d^	–
Respiratory status^e^	Room air and no artificial support or aids	Requires frequent suctioning of the nose or mouth and/or requires supplemental oxygen	Frequently has difficulty in breathing WITHOUT color change	Frequently has difficulty in breathing WITH color change	Frequently stops breathing and someone has to stimulate him or her to remind him or her to breath

a *Addition of insomnia to mild dysfunction in the mental status domain*.

b *Adapted feeding mild dysfunction to focus on signs of aspiration rather than requirement for assistance with feeding*.

c *Moderate feeding dysfunction no longer includes need for tube feeding*.

d *Severe dysfunction adapted to include children requiring tube feeds and parenteral nutrition removed from the definition*.

e *Respiratory domain scores adapted to remove use of devices such as tracheostomy, positive pressure, or mechanical ventilation originally defined as moderate, severe, and very severe dysfunction, respectively*.

The primary outcome was decline in functional status, defined as a change of ≥3 in the cumulative FSS score between baseline and 28-day follow-up. Secondary outcome measures included new domain morbidity, defined as a change of ≥2 in any individual FSS domain (9, 11, 12, 25), any decline in FSS from the baseline score, the cumulative 28-day FSS score, and the hospital length-of-stay (LOS). At 28-day follow-up, guardians were also asked whether their child was back to their pre-illness baseline state of health, and if not recovered, they were asked to describe what was different.

### Statistical Analyses

All data were collected by research personnel from multiple sources including the electronic medical record, paper chart, providers, and guardians. Study data were collected and managed using REDCap (Research Electronic Data Capture, version 7.2.2), a secure, web-based application designed to support data capture for research studies, hosted at MNH (33). All data were deidentified prior to analysis. Data were analyzed and descriptive statistics were performed using Stata Version 16 (StataCorp, College Station, TX). This included means and standard deviations, medians and interquartile ranges (IQR), counts, and percentages as required based on the type of data and its distribution.

Data analysis was performed on subjects alive at 28-day follow-up and with primary outcome data (baseline and follow-up FSS scores) complete. Using descriptive statistics, we describe the patient and clinical characteristics at the time of presentation for this cohort. We also determined the frequency, distribution, median, and IQR of cumulative FSS scores and of individual domains, as well as the median change in FSS score between the two time points. Bivariate analyses testing potential risk factors for decline in functional status were performed using Pearson chi-squared and Wilcoxon rank-sum tests. A *p-*value < 0.05 was considered statistically significant. The ability of the FSS to predict those patients identified by their guardians as not having recovered to their pre-illness baseline was evaluated by comparing positive and negative predictive values (NPV). A multivariable logistic regression model was then completed to further assess potential risk factors for a decline in function. Preidentified potential risk factors or confounders were included in the regression model, as well as variables identified as statistically significant (*p* < 0.05) on bivariate analysis.

## Results

**Figure 1:**
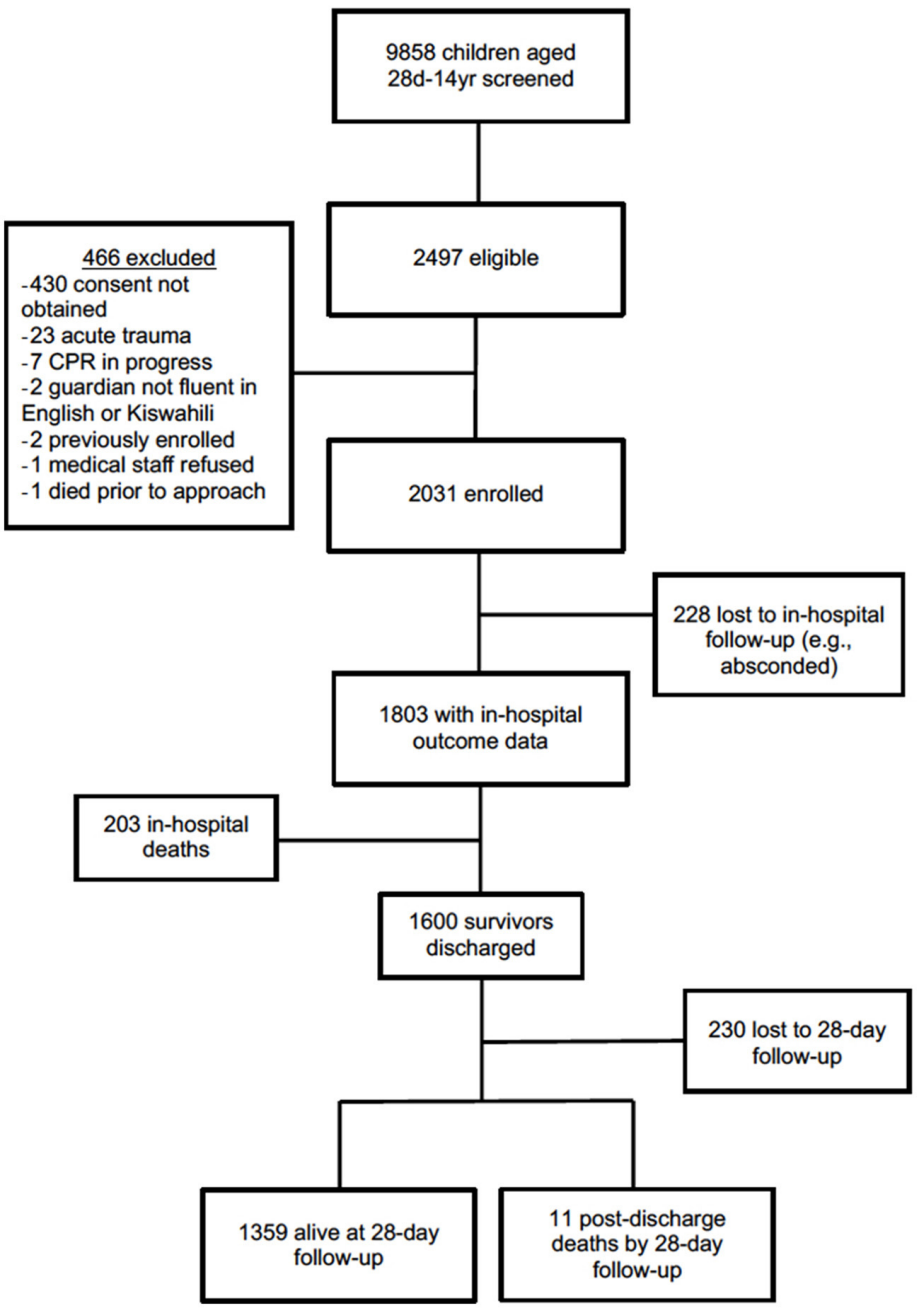
Study cohort flowchart including screening, enrollment, and outcomes.

The study cohort consisted of 2,031 total subjects enrolled with 1,359 alive at 28-day follow-up and with outcome data available (Figure 1). This cohort of survivors had a median age of 26.8 months (IQR 14.1, 55.3), of which 18.6% were ≤ 12 months old and 76.4% were ≤ 5 years old (Table 2). Malnutrition was common; 9.4% (128/1,358) were underweight and 12.1% (164/1,358) were malnourished. Half of patients (43.4%, 505/1,163) had a delayed time to presentation defined as fever duration >2 days prior to presenting to MNH and 39.4% (534/1,357) of patients were referred from another hospital (Table 1). One-tenth of patients (7.8%, 106/1,357) were hypoxic (SpO_2_ <92% in room air) upon presentation, 5.8% (79/1,359) had decreased responsiveness as defined by a score of verbal or worse on the AVPU (alert, verbal, pain, unresponsive) scale, and 47.3% (643/1,359) exhibited at least one warning sign using the LODS (Table 2). There was significant overlap between those exhibiting hypoxia, altered responsiveness, and organ dysfunction; the majority of the cohort [94% (1,277/1,359)] did not display any of these three worrisome clinical signs.

**Table 2:** Baseline characteristics at the time of presentation for 1,359 patients with 28-day follow-up data, and a bivariate comparison of baseline characteristics by change in functional status.

**Patient characteristic**	**Total (*N* = 1,359)**	**Functional status unchanged (*N* = 1,301)**	**Decline in functional status (*N* = 58)**	***P*-value**
Age (months), median (IQR)	26.8 (14.1–55.3)	26.8 (14.4–55.0)	26.3 (9.5–73.9)	0.79
Age categories, *n* (%)				0.09
0–12 months	253 (18.6)	237 (18.2)	16 (27.6)	
12–24 months	785 (57.8)	760 (58.5)	25 (43.1)	
2–5 years	266 (19.6)	253 (19.5)	13 (22.4)	
5+ years	54 (4.0)	50 (3.8)	4 (6.9)	
Male sex, *n* (%)	780 (57.4)	747 (57.4)	33(56.9)	0.94
WFAZ category, *n* (%)				**<0.001**
Severely underweight	164/1,358 (12.1)	144/1,300 (11.1)	20 (34.5)	
Underweight	128/1,358 (9.4)	124/1,300 (9.5)	4 (6.9)	
Average weight	684/1,358 (50.4)	669/1,300 (51.5)	15 (25.9)	
Medical history, *n* (%)				
HIV positive	11/151 (7.3)	10/141 (7.1)	1/10 (10.0)	0.74
History of CHD	116/1,353 (8.6)	101/1,295 (7.8)	15 (25.9)	**<0.001**
History of cancer	19/1,344 (1.4)	18/1,286 (1.4)	1 (1.7)	0.83
Immunization status, *n* (%)				0.67
Fully vaccinated	1,339/1,357 (98.7)	1,281/1,299 (98.6)	58 (100.0)	
Incompletely/not vaccinated	13/1,357 (1.0)	13/1,299 (1.0)	0	
Unknown status	5/1,357 (0.4)	5/1,299 (0.4)	0	
Maternal education level, *n* (%)				**0.01**
No formal school	49/1,356 (3.6)	45/1,298 (3.5)	4 (6.9)	
Primary school	608/1,356 (44.8)	571/1,298 (44.0)	37 (63.8)	
Secondary school	336/1,356 (24.8)	326/1,298 (25.1)	10 (17.2)	
University/Advanced degree	348/1,356 (25.7)	341/1,298 (26.3)	7 (12.1)	
Unknown	15/1,356 (1.1)	15/1,298 (1.2)	0 (0.0)	
Duration of illness, *n* (%)				0.10
Fever ≤ 2 days	658/1,163 (56.6)	629/1,121 (56.1)	29/42 (69.0)	
Fever >2 days	505/1,163 (43.4)	492/1,121 (43.9)	13/42 (31.0)	
Referral status, *n* (%)				0.41
Walk-in	823/1,357 (60.6)	790/1,299 (60.8)	33 (56.9)	
Referred from clinic/hospital	534/1,357 (39.4)	509/1,299 (39.2)	25 (43.1)	
Received antibiotics prior to arrival, *n* (%)	207/531 (39.0)	197/506 (38.9)	10/25 (40.0)	0.91
AVPU category: alert, *n* (%)	1,280 (94.2)	1,231 (94.6)	49 (84.5)	**0.001**
LODS, *n* (%)				0.92
No warning signs	716 (52.7)	684 (52.6)	32 (55.2)	
1 warning sign	523 (38.5)	501 (38.5)	22 (37.9)	
2 warning signs	115 (8.5)	111 (8.5)	4 (6.9)	
3 warning signs	5 (0.4)	5 (0.4)	0 (0.0)	
Heart rate abnormal for age, *n* (%)	710 (52.3)	672/1,300 (51.7)	38 (65.5)	**0.04**
Respiratory rate abnormal for age, *n* (%)	1,165/1,358 (85.8)	1,116/1,300 (85.8)	49 (84.5)	0.77
Hypoxic (O_2_ <92%), *n* (%)	106/1,357 (7.8)	95/1,299 (7.3)	11 (19.0)	**0.001**

*IQR, interquartile range; WFAZ, weight-for-age z-score; CHD, congenital heart disease; HIV, human immunodeficiency virus; Severely Underweight: WFAZ < −3; Underweight: WFAZ −2 to −3; average weight: WFAZ −2 to 2; HIV positive by either antigen testing, antibody testing, or PCR testing during admission; AVPU, alert-verbal-painful-unresponsive stepwise scale of consciousness; LODS, Lambaréné organ dysfunction score; O_2_, oxygen saturation. Bold values indicate significant findings with a p-value <0.05*.

Excluding subjects that were lost to follow-up, in-hospital mortality occurred in 11.3% (203/1,803) of subjects and an additional 0.8% (11/1,370) experienced post-discharge mortality by 28 days (Figure 1). Median hospital LOS among survivors at 28-days was 3 days (IQR 0.0–10.0).

**Figure 2:**
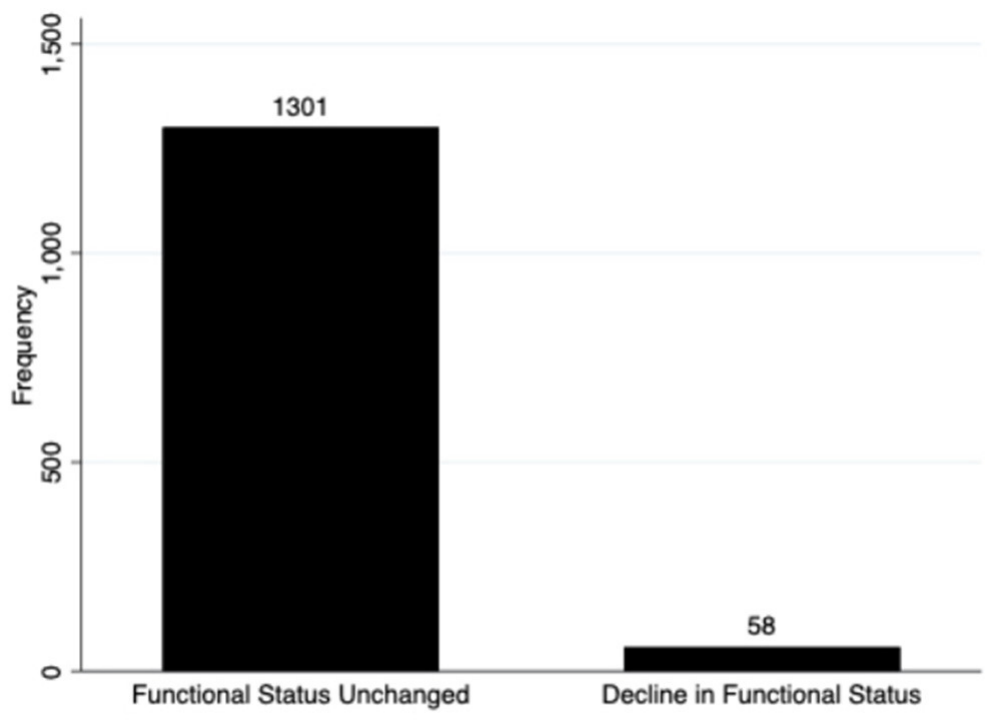
Frequency of the primary outcome, a decline in functional status.

**Figure 3:**
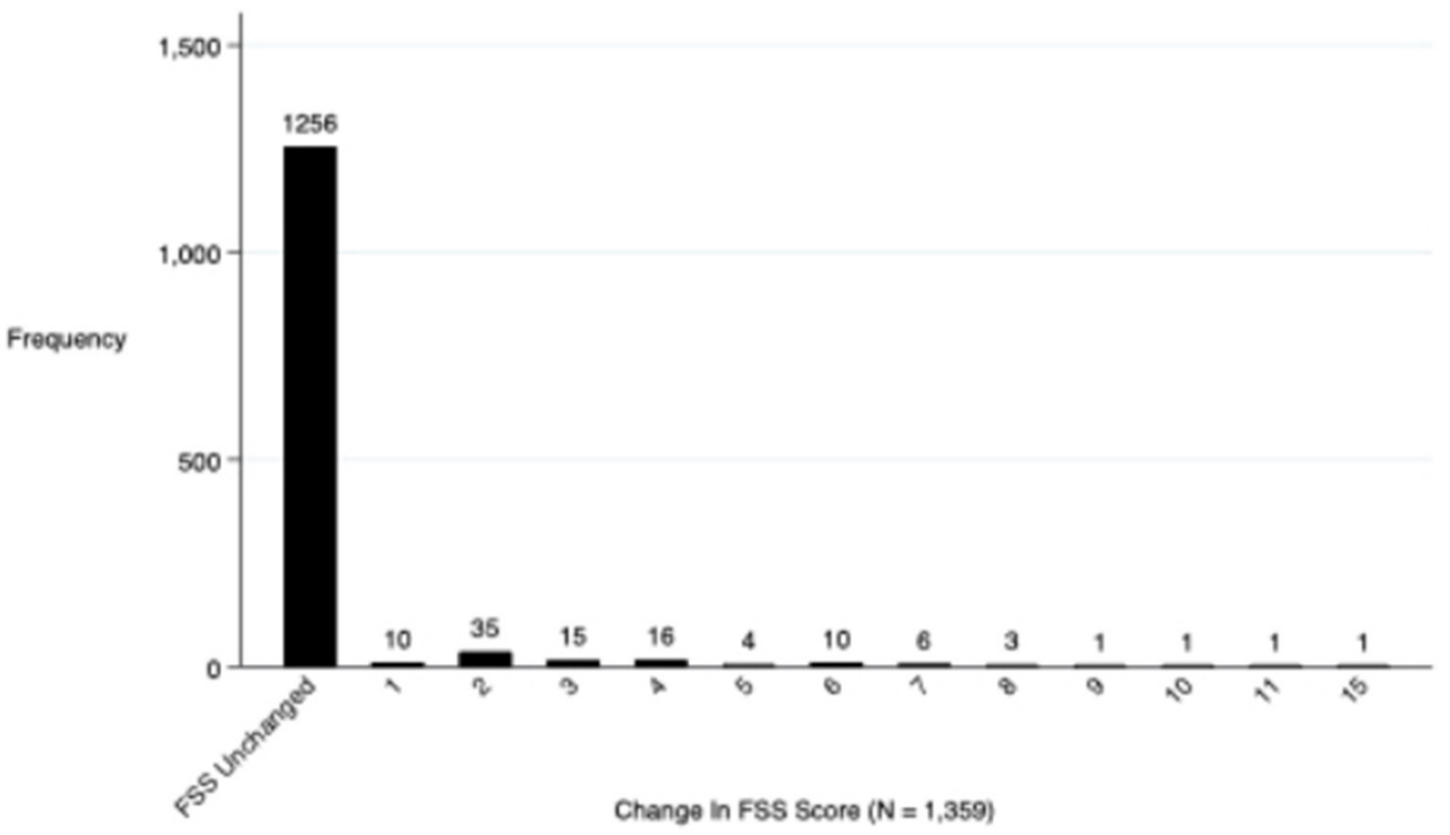
Frequency of changes in subject's FSS scores between baseline and 28-day follow-up.

**Figure 4:**
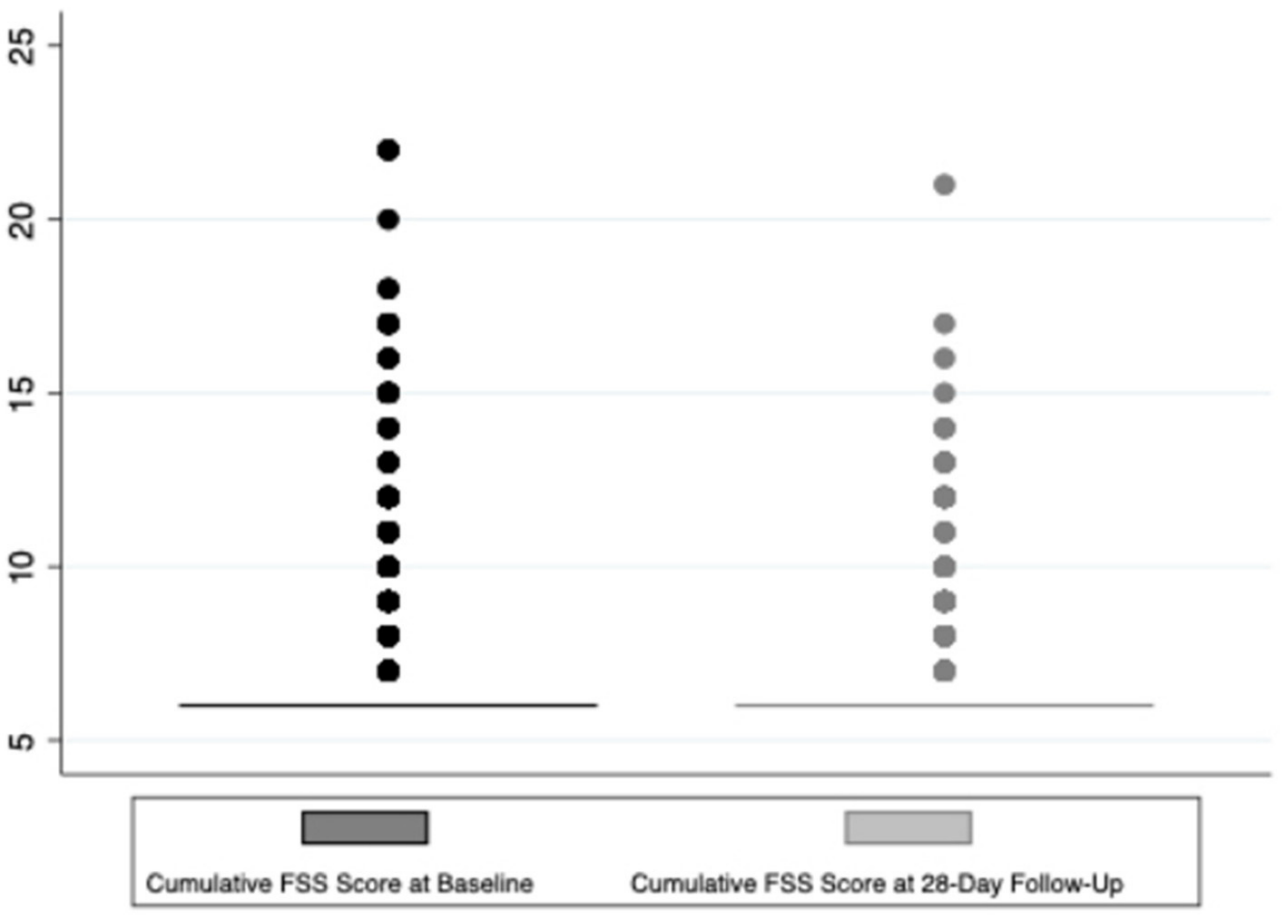
Box plot of baseline cumulative FSS score and 28-day follow-up FSS score.

Regarding the primary outcome, 4.3% (58) of the 1,359 surviving children who completed 28-day follow-up had a decline in functional status defined as a change of ≥3 in the cumulative FSS score between baseline and 28-day follow-up (Figure 2). Additionally, a total of 7.5% (103/1,359) of these subjects showed any decline in their FSS score from baseline to 28-day follow-up (a change in FSS score of at least one) (Figure 3). Of these 1,359 survivors, cumulative FSS score at the 28-day follow-up showed that 91.1% of children had “normal function,” 4.8% had “mild dysfunction,” 3.9% had “moderate dysfunction,” 0.2% had “severe dysfunction,” and no children had “very severe dysfunction.” Both baseline and 28-day cumulative FSS scores had a median of 6, consistent with most subjects having “normal function” at the time of both assessments (Figure 4). Among individual domains, motor function, feeding, and respiratory status most commonly demonstrated a decline in functional status (3.4, 3.8, and 2.1%, respectively) (Table 3).

**Table 3:** Secondary outcome measures for patients surviving at 28-day follow-up.

**Secondary outcome**	
Hospital LOS in survivors, median (IQR)	3.0 (0.0, 10.0)
Subjectively not recovered to baseline at 28-day follow-up, *N* (%)	191/1,380 (13.8%)
**Cumulative FSS score category at 28-day follow-up in survivors**, ***N*** **(%)**	
Normal functional status	1,238/1,359 (91.1%)
Mildly impaired functional status	65/1,359 (4.8%)
Moderately impaired functional status	53/1,359 (3.9%)
Severely impaired functional status	3/1,359 (0.2%)
Very severely impaired functional status	0/1,359 (0%)
**Decline in FSS domain function from baseline to 28-day follow-up in survivors**, ***N*** **(%)**	
Mental status	12/1,359 (0.9%)
Sensory	3/1,359 (0.2%)
Communication	3/1,359 (0.2%)
Motor function	46/1,359 (3.4%)
Feeding	51/1,359 (3.8%)
Respiratory	28/1,359 (2.1%)

*LOS, length of stay; IQR, interquartile range; Subjective recovery to baseline determined by asking guardian if the child was back to pre-illness baseline; FSS, functional status scale; normal functional status: FSS score 6–7; mildly impaired functional status: FSS score 8–9; moderately Impaired Functional Status: FSS score 10–15; severely impaired functional status: FSS score 16–21; very severely impaired functional status: FSS score >21; decline in FSS domain function: domain score difference of ≥2*.

Regarding guardian-reported recovery at 28-days, 13.8% (191/1,380) of guardians reported that their child was not yet back to their pre-illness state (Table 3). Generalized weakness, pain, seizures, recurrent illness and fever, vomiting, feeding intolerance, and poor appetite were the most frequently reported reasons that the child had not recovered to baseline, none of which are factors captured by the adapted FSS. The adapted FSS accurately identified a new morbidity 56% (32/57) of the time when compared to the caregiver's report of recovery (positive predictive value, PPV). Conversely, the adapted FSS correctly identified children with no change in functional status 89% (1,153/1,291) of the time when compared to the guardian's report of recovery (NPV). The PPV and NPV of the FSS for identifying children not recovered to their pre-illness baseline remained unchanged [56% (57/101) and 91% (1,134/1,237), respectively] when including subjects with any decline in their FSS score as opposed to only those with a decline of at least three points.

In survivors who completed 28-day follow-up, the guardian present at time of admission was the mother 82% of the time (1,113/1,359), the father 11% of the time (151/1,359), and other family members or a neighbor 7% of the time (95/1,359). Conversely, at 28-day follow-up, the vast majority (>98%) of FSS were completed by one of the two parents [78.7% (1,070/1,359) mother; 19.3% (263/1,359) father]. The same guardian completed both FSS evaluations 72.3% of the time (983/1,359). When restricting the outcome analysis to subjects who had both FSS questionnaires completed by the same guardian or by the same one of their parents, there was still approximately 4% (39/983 and 39/977, respectively) of subjects with a decline in functional status.

Risk factors for a decline in functional status as identified by the adapted FSS using bivariate analysis included presence of malnutrition (*p* <0.001), having a history of congenital heart disease (*p* <0.001), a lower maternal education level (*p* = 0.011), decreased level of consciousness as indicated by a score of verbal or worse on the AVPU scale (*p* = 0.001), abnormal heart rate for age (*p* = 0.04), and hypoxia at presentation (*p* <0.001) (Table 2). On further assessment of potential risk factors utilizing a multivariable logistic regression model, abnormal heart rate for age [odds ratio (OR) 2.62, 95% confidence interval (CI) 1.24–5.53] and a decreased level of responsiveness as defined by the AVPU scale (OR 3.35, 95% CI 1.28–8.74) were associated with an increased odds of a decline in functional status. Normal nutritional status using weight-for-age z-score (OR 0.21, 95% CI 0.09–0.49) and mothers with university-level/advanced degrees (OR 0.13, 95% CI 0.03–0.71) had decreased odds of a decline in functional status (Table 4).

**Table 4:** Multivariable logistic regression model results: unadjusted and adjusted odds of decline in functional status.

**Patient characteristic variable**	**Unadjusted OR**	**Unadjusted 95% CI**	**Adjusted OR**	**Adjusted 95% CI**
Male sex	0.98	(0.58–1.67)	1.21	(0.60–2.43)
Age categories				
0–11 months old	Reference		Reference	
12–60 months old	**0.49**	**(0.26–0.93)**	0.75	(0.32–1.80)
5–12 years old	0.76	(0.36–1.62)	0.57	(0.10–3.14)
12–14 years old	1.12	(0.38–3.70)	0.34	(0.03–4.44)
Nutritional status by WFAZ category				
Severely underweight	Reference		Reference	
Underweight	**0.23**	**(0.08–0.70)**	**0.14**	**(0.03–0.64)**
Average weight	**0.16**	**(0.08–0.32)**	**0.21**	**(0.09–0.49)**
Overweight	**0.38**	**(0.20–0.73)**	0.37	(0.08–1.69)
History of CHD	**4.14**	**(2.23–7.72)**	1.96	(0.73–5.29)
Maternal education level				
None	Reference		Reference	
Primary school	0.73	(0.25–2.14)	0.46	(0.12–1.79)
Secondary school	0.35	(0.10–1.15)	0.23	(0.05–1.06)
University/advanced degree	**0.23**	**(0.07–0.82)**	**0.13**	**(0.03–0.71)**
Fever duration >2 days	0.57	(0.29–1.11)	**0.47**	**(0.22–0.97)**
Abnormal heart rate for age	**1.77**	**(1.02–3.08)**	**2.62**	**(1.24–5.53)**
Hypoxic (O_2_ <92%)	**2.97**	**(1.49–5.91)**	0.45	(0.12–1.74)
Not alert on presentation	**3.23**	**(1.53–6.84)**	**3.35**	**(1.28–8.74)**
Hospital length of stay (Days)	1.01	(1.00–1.02)	1.01	(1.00–1.02)

*OR, odds ratio; CI, confidence interval; WFAZ, weight-for-age z-score; severely underweight: WFAZ < −3; underweight: WFAZ −2 to −3; average weight: WFAZ −2 to 2; overweight: WFAZ >2; CHD, congenital heart disease; patients were assessed for alertness at presentation using the AVPU (alert-verbal-painful-unresponsive) scale of level of consciousness; O_2_, oxygen saturation. Bold values indicate significant findings with a p-value <0.05 and a 95% confidence interval not crossing 1*.

## Discussion

In this cohort of pediatric sepsis survivors, we identified a low proportion of subjects with a decline in functional status compared to their pre-illness baseline, with only 4.3% of surviving children demonstrating a change in FSS score ≥3. A total of 7.5% of children demonstrated any decline in their FSS with a worsening score by at least one point from baseline to 28-day follow-up. The individual domains most impacted were motor function, feeding, and respiratory status. Risk factors for poor functional outcomes and recovery included presenting with an abnormal heart rate or a decreased level of responsiveness, while having a healthy nutritional status and a high maternal education level were factors associated with a good functional outcome. Lastly, children were far more likely to be identified by their guardians as not being recovered to their pre-illness baseline than to have a decline in functional status as defined by the FSS (13.8 vs. 4.3%).

Prior studies utilizing the FSS to assess pediatric outcomes following acute septic episodes or other critical illnesses have been conducted in resource-rich settings, and often demonstrate a much higher proportion (up to 50%) of new functional morbidity and functional decline (8, 9, 11, 12, 26, 27). Pereira et al., conducted a study of functional outcomes in children after admission to a PICU in Brazil and demonstrated a moderate impairment in function upon PICU discharge, with motor function and feeding domains most commonly impacted (8). However, the Brazilian study was unable to determine baseline functional status or the frequency of decline. Furthermore, both the studies conducted in resource-rich settings and in Brazil included only children who developed critical illness secondary to sepsis, indicating a higher severity of illness than this Tanzanian cohort. Thus, it is difficult to compare results as our cohort is inclusive of all children presenting with an acute pediatric sepsis episode, rather than restricting the patient population to only those who develop critical illness and severe sepsis or septic shock. It is therefore not surprising that the functional outcomes in the Tanzanian cohort are better overall than those in more severely ill cohorts.

The FSS domains most frequently affected in this Tanzanian cohort were feeding, motor function, and respiratory status, which is consistent with studies assessing functional outcomes following pediatric critical illnesses. Interestingly, the domains most commonly impacted following the septic episode were not concordant with those that had the highest levels of baseline dysfunction, which included mental status, motor, and respiratory. This impact of pediatric sepsis on feeding, respiratory, and motor function suggests that there may be a need for outpatient speech therapy and physical therapy following acute illness in Tanzanian pediatric sepsis survivors to optimize long-term outcomes. These therapy services are often not readily available, suggesting an area of opportunity for future investment and improvement in care following hospital discharge.

While there was an overall low proportion of poor functional outcomes as identified by the FSS in this cohort, a much higher proportion of children (13.8%, 191/1,380) were subjectively identified by their guardians as not being recovered to baseline at 28 days. Several factors important to QOL including persistent generalized weakness, pain, seizures, recurrent illness, vomiting, feeding intolerance, and poor appetite were commonly identified as reasons a child had not returned to their pre-illness baseline. This discrepancy, along with poor PPV of 56% for identifying new morbidity in pediatric sepsis survivors suggests that the FSS has limitations in this population, despite being adapted for a resource-limited setting. The FSS may not adequately capture these new morbidities that are impactful on a child's QOL and ability to function independently, and it is important that these factors be included with future assessments of functional status.

One consideration regarding the limitations of the FSS in this study was that defining a decline in functional status as an FSS change of ≥3 was too stringent, and that parents may have noticed more subtle changes in their child's function from baseline. Additional analysis was performed to assess the concordance between guardian's subjective report of recovery and any decline in FSS score from baseline, defined as a change in FSS score of at least one point. This additional analysis demonstrated no change in the concordance between the subjective report of recovery and the FSS, revealing the same PPV of 56% (57/101) and a similar NPV of 91% (1,134/1,237). This suggests that there were true limitations in the ability of the FSS, even when adapted, to detect meaningful changes in a child's functional status in this cohort. Furthermore, the lack of a clear gold standard tool to validate the FSS and its ability to accurately identify patients with a new morbidity, particularly in resource-limited settings, remains an important limitation. The utilization of other functional or QOL scores as a validation tool was not possible with the data available in this cohort due to the resource-limited setting.

Based on previously published literature, we identified comorbidities (malnutrition, congenital heart disease), socioeconomic factors (maternal education), clinical signs (hypoxia, altered mental status), and hospital LOS as potential risk factors for worse functional outcomes (8, 12, 14, 17). Our analysis identified nutrition status, maternal education level, and signs of increased severity of illness on presentation, including abnormal heart rate for age and decreased level of consciousness, as risk modifiers of a child's post-sepsis functional status. It is important that these risk factors for worse functional outcomes are identified as this may allow for locations with limited therapy (i.e., physical, occupational, or speech) and rehabilitation resources to allocate those therapies to patients at highest risk for poor functional outcomes. However, our ability to assess the impact of comorbidities, such as history of HIV, congenital heart disease, or cancer, on functional outcomes was very limited due to incomplete data.

There were several limitations to our study. While we had a low number of surviving patients lost to follow-up (230/1,600, 14.4%), a sensitivity analysis conducted to assess baseline characteristics of those lost to follow-up did show a few statistically significant variations from those with outcome data (Supplementary Table 1). These included multiple variables identified as risk factors for worse functional outcomes, including level of consciousness, maternal education level, and nutritional status, suggesting that our outcome data may underestimate those with poor outcomes, including mortality and a decline in functional status.

Another limitation of our study was that over 25% of the FSS were completed by two different people at baseline and follow-up, increasing the likelihood of subjective variation between guardians. Additionally, there was a higher percentage of FSS completed by someone other than the mother or father at admission, compared to follow-up, which may have contributed to less accurate baseline FSS scores with improvement in some scores (approximately 16.6% of subjects) on follow-up. However, when accounting for this and restricting outcome data analysis to only those with both FSS scores completed by the same guardian or the same parent, the overall rate of a decline in functional status remained unchanged and low at approximately 4% of subjects. Furthermore, the language barrier and the translatability of the FSS and other universal scoring systems to Kiswahili, a primary language of Tanzania, may have contributed to misinterpretation when asked about a child's pre-illness baseline level of function, leading guardians to include impacts of the acute illness in their baseline assessments and creating an artificially decreased baseline functional score.

An additional limitation was the brevity of the follow-up of our patients, which was limited to 28 days following initial presentation. Prior studies of pediatric sepsis survivors with longer follow-up intervals demonstrate variable improvement in functional status over time, with resolution of “new morbidity” or “new disability” in up to 65–95% of patients at 1-year (9, 18). However, most of these studies with extended periods of follow-up have been conducted in resource-rich settings, where therapies are routinely available post-discharge, allowing for optimal outcomes to be achieved. One study, conducted in India, reported that only 5% of those with a “new disability” at PICU discharge did not have recovery by 1-year follow-up. However, this study was limited by a small sample size and high (26%) mortality with only 84 of their initial 121 patients completing 1-year follow-up (18). Conversely, in a study conducted across 12 academic PICUs in the United States, health-related QOL among one-third of children with pre-existing severe developmental disabilities had not recovered to baseline at 1-year follow-up. This indicates that a baseline developmental disability may be a significant risk factor for long-lasting new morbidity secondary to sepsis, which we were unable to assess in this study (15).

Furthermore, our study utilized the LODS as a severity of illness score, but there are no data on the ability of the LODS to predict new functional morbidity following pediatric sepsis. Several pediatric illness severity scores, including the Pediatric Sequential Organ Failure Assessment (pSOFA) and the PEdiatric Logistic Organ Dysfunction (PELOD-2) score, and the Pediatric Risk of Mortality (PRISM III) score, have been shown to help distinguish patients likely to have new morbidity at discharge (11, 19, 28). However, these scores are often not possible to calculate or determine in settings with limited resources where only clinical data are readily available, rather than biochemical or laboratory data, or more complex physiologic data. Lastly, our study excluded patients who arrived in cardiac arrest, which may be another limitation, as receiving CPR has been identified as a risk factor for worse functional outcomes (18, 19).

There were several notable strengths to this study as well. First, this study had a large sample size, with over 2,000 subjects initially enrolled. Excluding the patients who died during the 28-day duration of this study, there was an overall low percentage of subjects lost to follow-up despite the logistical difficulties inherent to conducting a pediatric cohort study in an LMIC. This study had over 1,350 patients (74.8% of the surviving cohort) with comprehensive 28-day follow-up data available, including functional outcomes. Additionally, to the best of our knowledge, this study was the first to utilize an adapted and translated form of the FSS to assess functional outcomes in children. These adaptations focused primarily on removing impertinent sections of the original FSS that are not applicable to resource-limited settings, such as a reliance on only parenteral nutrition or having a tracheostomy with ventilator-dependence. However, there were also initial adaptations made to include factors likely to be important in a child's day to day function and QOL, such as the addition of insomnia to mental status dysfunction. These original adaptations were novel and allowed for the use of the FSS in a resource-limited setting, where it is often difficult or impossible to use available tools to assess functional or QOL outcomes.

We propose that several additional changes be made to the adapted FSS to improve its use in assessing functional outcomes in resource-limited settings. These changes would include adding: “new, recurrent seizures” to the “mild dysfunction” category of the mental status domain; “new, persistent pain” to the “mild dysfunction” category of the sensory function domain; “generalized weakness” to the “mild dysfunction” category of the motor functioning domain; and “feeding intolerance, vomiting, and poor appetite” to the “mild dysfunction” category of the feeding domain. This would aim to capture the vast majority of functional and QOL-related reasons that guardians identified for a child not returning to their pre-illness state of health and function. These proposed revisions to the adapted FSS for resource-limited settings will require internal validation within our dataset, as well as further study and external validation in other cohorts and settings.

## Conclusions

Significant strides have been made in the reduction of sepsis-related mortality, but with that comes a pressing need to appreciate morbidity in pediatric sepsis survivors, and to learn how to best reduce the development of these impactful morbidities. This study in Tanzanian pediatric sepsis survivors identified a relatively low percentage of children with significant functional decline in comparison to other studies. However, a larger percentage of patients were identified as subjectively not having recovered to their pre-illness baseline due to factors important to a child's QOL and ability to function. The FSS requires further adaptation for resource-limited settings to optimally capture children with a functional decline following an acute septic episode, and to better identify follow-up needs upon discharge. Additionally, there is a need for further studies with a longer duration of follow-up to better understand long-term functional outcomes following pediatric sepsis.

## Data Availability Statement

The raw data supporting the conclusions of this article will be made available by the authors, without undue reservation.

## Ethics Statement

The studies involving human participants were reviewed and approved by the Institutional Review Boards and Committees on Human Research at both Muhimbili University of Health and Allied Sciences (Ref. No. 2016-03-30/AEC/Vol.X/201) and the University of California, San Francisco (IRB # 16-18977, Ref. No. 161295). Written informed consent to participate in this study was provided by the participants' legal guardian/next of kin.

## Author Contributions

TK, HS, BM, and MM: conception and design of the work. TK, HS, and BM: data acquisition. SL-B: data analysis and first draft of the manuscript. SL-B, TK, MS, and AS: data interpretation. SL-B, TK, MM, BM, MS, and AS: manuscript revision and editing. SL-B, MS, TK, HS, MM, BM, TR, and AS: final approval of the version to be published and agreement to be accountable for all aspects of the work.

## Funding

Research effort to create this publication was supported by the National Institute of Allergy and Infectious Diseases (award number K23AI144029, TK) of the National Institutes of Health (NIH), the University of California, San Francisco (UCSF) Division of Critical Care, the UCSF Department of Pediatrics Clinical-Translational Pilot grant, and the UCSF Resource Allocation Program Pilot for Junior Investigators in Basic and Clinical/Translational Sciences. The funders had no role in study design, data collection and analysis, decision to publish, or preparation of the manuscript.

## Author Disclaimer

The views expressed are those of the authors and not necessarily those of the NIH or UCSF.

## Conflict of Interest

The authors declare that the research was conducted in the absence of any commercial or financial relationships that could be construed as a potential conflict of interest.

## Publisher's Note

All claims expressed in this article are solely those of the authors and do not necessarily represent those of their affiliated organizations, or those of the publisher, the editors and the reviewers. Any product that may be evaluated in this article, or claim that may be made by its manufacturer, is not guaranteed or endorsed by the publisher.
