# Supplementary material for: Functional Outcomes and Morbidity in Pediatric Sepsis Survivors: A Tanzanian Experience
Source: Front Pediatr. 2022 Jan 17;9:805518. doi: 10.3389/fped.2021.805518 (PMC8801911; doi:10.3389/fped.2021.805518)
Supplement: Supplementary file 1 [file Table_1.DOCX]

| Supplemental Table 1 \| Baseline patient characteristics comparing patients alive at 28-day follow up to patients lost to 28-day follow up | | | |
| --- | --- | --- | --- |
| Patient Characteristic | **Total Alive at 28-Day Follow-Up**  (N=1359) | **Patients Lost to 28-Day Follow-Up**  (N=230) | **p-value** |
| Age (months), median (IQR) | 26.8 (14.1 – 55.3) | 21.5 (12.0 – 49.9) | **0.01** |
| Age Categories, n(%)  0-12 months  12-24 months  2-5 years  5+ years | 253/1357 (18.6)  350/1357 (25.8)  435/1357 (32.1)  319/1357 (23.5) | 55 (23.9)  68 (29.6)  57 (24.8)  50 (21.7) | 0.08  0.26  **0.03**  0.62 |
| Male Sex, n(%) | 780 (57.4) | 136 (59.1) | 0.67 |
| WFAZ Category, n(%)  Severely Underweight  Underweight  Average Weight | 164/1358 (12.1)  128/1358 (9.4)  684/1358 (50.4) | 44/207 (21.3)  31/207 (15.0)  129/207 (62.3) | **<0.001**  **0.02**  **<0.01** |
| Medical History, n(%)  HIV Positive  History of CHD  History of Cancer | 11/151 (7.3)  116/1353 (8.6)  19/1344 (1.4) | 4/35 (11.4)  20 (8.7)  4 (1.7) | 0.64  **<0.001**  **0.01** |
| Immunization Status, n(%)  Fully Vaccinated  Incompletely/Not Vaccinated  Unknown Status | 1339/1357 (98.7)  13/1357 (1.0)  5/1357 (0.4) | 226 (98.3)  3 (1.3)  1 (0.4) | 0.85  0.90  1.00 |
| Maternal Education Level, n(%)  No Formal School  Primary School  Secondary School  University/Advanced Degree  Unknown | 49/1356 (3.6)  608/1356 (44.8)  336/1356 (24.8)  348/1356 (25.7)  15/1356 (1.1) | 22 (9.6)  127 (55.2)  51 (22.2)  28 (12.2)  2 (0.9) | **<0.001**  **<0.01**  0.44  **<0.001**  1.00 |
| Duration of Illness, n(%)  Fever <=2 Days  Fever >2 Days | 658/1163 (56.6)  505/1163 (43.4) | 79/182 (43.4)  103/182 (56.6) | **<0.01**  **<0.01** |
| Referral Status, n(%)  Walk-in  Referred from Clinic/Hospital | 823/1357 (60.6)  534/1357 (39.4) | 95 (41.3)  135 (58.7) | **<0.001**  **<0.001** |
| Received Antibiotics Prior to Arrival, n(%) | 207/531 (39.0) | 59/135 (43.7) | 0.37 |
| AVPU Category: Alert, n(%) | 1280 (94.2) | 189 (82.2) | **<0.001** |
| LODS, n(%)  No Warning Signs  1 Warning Sign  2 Warning Signs  3 Warning Signs | 716 (52.7)  523 (38.5)  115 (8.5)  5 (0.4) | 104 (45.2)  101 (43.9)  22 (9.6)  3 (1.3) | **0.04**  0.14  0.67  0.18 |
| Heart Rate Abnormal for Age, n(%) | 710 (52.3) | 127/230 (55.2) | 0.41 |
| Respiratory Rate Abnormal for Age, n(%) | 1165/1358 (85.8) | 188/230 (81.7) | 0.09 |
| Hypoxic (O_2_ < 92%), n(%) | 106/1357 (7.8) | 34/230 (14.8) | 0.08 |
| *IQR, interquartile range; WFAZ, weight-for-age z-score; CHD, congenital heart disease, HIV, Human Immunodeficiency Virus; Severely Underweight: WFAZ < -3; Underweight: WFAZ -3 to -2; normal weight: WFAZ -2 to +2; AVPU, Alert-Verbal-Painful-Unresponsive stepwise scale of consciousness; LODS, Lambaréné Organ Dysfunction Score; O_2_, Oxygen saturation* | | | |
